# The Potential of Targeting Brain Pathology with Ascl1/Mash1

**DOI:** 10.3390/cells6030026

**Published:** 2017-08-23

**Authors:** Bor Luen Tang

**Affiliations:** 1 Department of Biochemistry, Yong Loo Lin School of Medicine, National University of Singapore, Singapore 117596, Singapore; bchtbl@nus.edu.sg; Tel.: +65-6516-1040; 2 NUS Graduate School for Integrative Sciences and Engineering, National University of Singapore, Singapore 117596, Singapore

**Keywords:** Achaete-scute complex-like 1 (Ascl1), glioblastoma, stroke

## Abstract

The proneural factor Achaete-scute complex-like 1 (Ascl1/Mash1) acts as a pioneering transcription factor that initializes neuronal reprogramming. It drives neural progenitors and non-neuronal cells to exit the cell cycle, and promotes neuronal differentiation by activating neuronal target genes, even those that are normally repressed. Importantly, force-expression of Ascl1 was shown to drive proliferative reactive astroglia formed during stroke and glioblastoma stem cells towards neuronal differentiation, and this could potentially diminish CNS damage resulting from their proliferation. As a pro-neural factor, Ascl1 also has the general effect of enhancing neurite growth by damaged or surviving neurons. Here, a hypothesis that brain pathologies associated with traumatic/ischemic injury and malignancy could be targeted with pro-neural factors that drives neuronal differentiation is formulated and explored. Although a good number of caveats exist, exogenous over-expression of Ascl1, alone or in combination with other factors, may be worth further consideration as a therapeutic approach in brain injury and cancer.

## 1. Introduction

Central nervous system (CNS) pathologies due to traumatic or ischemic injury are often of devastating consequences, and the adult CNS cancer type glioblastoma has remained largely refractory to treatment despite intensive work on therapeutic interventions [1]. Although the pathological bases of injuries and malignancies do not appear to overlap significantly, both share a similar feature of unwanted proliferation of cells. In CNS injuries, astrocytes are reactivated by proinflammatory factors, and these reactivated astrocytes contribute to neuronal/glia demise and brain tissue scarring [2]. Glioblastomas contain a subpopulation of cancer stem cells [3] with neural precursor properties, and these highly renewable cells with lasting tumorigenic potential drive tumor progression continuously, and can survive therapeutic onslaughts. Intuitively, if one is able to stop the reactive astroglia and glioblastoma stem cells from active and excessive proliferation, much of their devastating effects could potentially be diminished. Developmentally speaking, these undesirable proliferations could be effectively halted if dividing cells are driven towards terminal differentiation. This intuition has a basis, as both astroglia and glioma cells could be potentially driven towards neuronal differentiation with the expression of the pro-neural factors.

Evolutionarily conserved pro-neural factors, including members of the basic helix-loop-helix (bHLH) class transcription factor and neurogenins, endow neural progenitor cells (NPCs) with a neuronal fate and drives neuronal differentiation [4,5]. Amongst these, Achaete-scute complex-like 1 (Ascl1), also known as Mammalian Achaete-Scute Complex Homolog 1 (Mash1), is considered a “master regulator” of neurogenesis in vertebrates [6,7]. Recent findings have shown that Ascl1 acts as a pioneering factor that initializes neuronal reprogramming and drives neuronal differentiation of multiple cell types, including reactive astroglia induced by stroke [8] and glioblastoma stem cells [9], thus effectively diminishing CNS damage resulting from their proliferation. In the paragraphs below, evidence in support of the hypothesis that exogenous over-expression of Ascl1 could be potentially explored as a therapeutic approach in CNS injury and malignancy will be examined.

## 2. Ascl1’s Neurogenic Activity and Its Role as a Direct Neural Reprogramming Factor

The pivotal role of Ascl1 in neurogenesis has been known for more than twenty years now [10]. Knockout of Ascl1 impaired neuron generation in multiple brain regions, while its over-expression in neural progenitor cells (NPCs) induced exit from the cell cycle and full neuronal differentiation. Interestingly, Ascl1 also appears to have a role in maintaining the proliferative state of NPCs, and this dual role could be down to its mode of expression. The level of Ascl1 in NPCs is repressed by Hairy and enhancer of split 1 (Hes1) [11] downstream of Notch signaling [12]. Hes1 levels oscillate in NPCs [13], and pro-neural genes like Ascl1 and Neurog2 under its regulation also exhibit an oscillation in their levels. Notably, Ascl1 is an unstable protein, and is actively degraded via polyubiquitination by the E3 ubiquitin ligase HUWE1/UREB1/MULE [14] through the proteasome. Upon neuronal induction, however, Ascl1 switches from the oscillatory mode to a sustained mode of expression. While the oscillatory Ascl1 expression maintains proliferating NPCs, sustained Ascl1 expression promotes neuronal differentiation [15]. The transcriptional activity of Ascl1 (and other pro-neural factors) is also cell cycle-dependent, and is regulated by cyclin-dependent kinases mediated phosphorylation, which attenuates its DNA binding [16,17]. These tight regulatory mechanisms contribute to the necessarily transient nature of pro-neural factor activities during neurogenesis.

A particularly interesting and important capacity of Ascl1 is its ability, either alone or in conjunction with other pro-neural factors, to directly reprogram various somatic cells into neurons [18]. The Götz lab has shown that cultured early postnatal cortical astroglia could be reprogrammed into electrophysiologically active neurons with Ascl1 and Neurogenin-2 (Neurog2) [19]. Other subsequent successes in converting astrocytes into neurons both in vitro and in vivo using Ascl1 in combination with other factors have been reported [8,20,21,22,23]. Wernig, Südhof and colleagues first demonstrated a direct conversion of mouse fibroblasts to induced neuronal (iN) cells capable of generating action potentials and synapse formation with three factors; namely, Ascl1, Brain 2 (Brn2/Pou3f2) and Myelin Transcription Factor 1-like (Myt1l) [24]. These same factors could also generate neurons from human fibroblast and stem cells [25], as well as terminally differentiated hepatocytes [26]. The authors subsequently showed that Ascl1 alone is sufficient to generate iNs from mouse/human fibroblast and stem cells (Chanda et al., 2014). A combination of Ascl1 and another proneural factor, Sex-determining region Y-box 2 (Sox2), was also shown to reprogram pericyte-derived cells of adult human brain into iNs [27]. The basis for this remarkable reprogramming ability of Ascl1 was revealed by its efficient occupancy of associated chromatin sites [28]. Ectopically expressed Ascl1 appears to be a pioneer transcription factor that binds quickly and readily to most of its cognate genomic sites in the fibroblast genome even when these are in a “closed” nucleosome-bound state, and may act to recruit Brn2 to these sites [28]. In fact, binding of Ascl1 was shown to precede increases in new regions of open chromatin, suggesting that its binding promotes an increase in chromatin accessibility during neurogenesis [6].

This direct neuronal reprogramming activity of Ascl1 thus allows for the following crude and simple-minded thought. The adult CNS consists largely of terminally differentiated neuronal and glia cells that no longer proliferate, which is a desirable state for the maintenance of its functional architecture. However, under pathological conditions, uncontrolled and undesirable cell divisions and proliferation occur, particularly during reactive glial responses in injury and in tumor growth, often with devastating consequences to neuronal survival and function. As terminally differentiated neurons would fully exit the cell cycle, a plausible way to curb uncontrolled and undesirable astroglia or tumor cell growth may be to simply turn them into neurons. In the following sections, evidence that this might be achievable with exogenous or forced Ascl1 expression, as well as the associated caveats of this approach, is discussed.

## 3. Ascl1 Converting Reactive Astrocytes into Neurons in Stroke

In traumatic or ischemic brain injuries, astrocyte reactivation underlies glial scar formation. These processes of astrogliosis and glial scarring serve to limit the spread of the pro-inflammatory factors released from lysed cells, but also contribute to neuronal death and inhibition of regeneration. Interestingly, however, an increase in Ascl1-expressing proliferating progenitor was observed by Zhang and colleagues in a right middle cerebral occlusion stroke model, and these cells eventually gave rise to GABAergic neurons and mature oligodendrocytes [29]. Frisén’s laboratory showed that stroke and reduced Notch signaling could elicit a latent neurogenic program with induced Ascl1 expression in striatal astrocytes, and this could produce new neurons [30]. Another interesting recent report from Nagy’s group showed that subventricular zone (SVZ) neural stem cells could migrate to ischemic injury sites in the cortex to give rise to reactive astrocytes. These SVZ neural stem cell-derived reactive astrocytes could be converted to neurons by forced over-expression of Ascl1 alone in vivo [8]. The conversion efficiency is not high, but interestingly, this could occur close to the lesion core where most of the endogenous neurons are dead or dying.

The above findings are in line with other reports that Ascl1 can drive conversion of astrocytes to neurons in vivo [21], and attests to the feasibility of using Ascl1 to suppress the detrimental effects of reactive gliosis by differentiating them into neurons. Similar astrocyte-neuron conversions have also been demonstrated in the injured cortex and striatum with Neurog2 [31], and the injured spinal cord with Sox2 [32]. It is notable that in neither of these two reports was Ascl1 shown to be effective for astrocyte-neuron conversion. This suggests that the effectiveness of Ascl1 in driving reactive neuronal differentiation of astroglia is dependent on the location of injury sites and their environments that host different astrocytic subpopulations.

## 4. Ascl1 Drives Neuronal Differentiation of Glioma and Glioblastoma Stem Cells

Glioblastomas are devastating brain cancers, as treatment of the primary tumor carries a high risk of endangering brain tissues, and these are often refractory to treatment because of the occurrence of cancer stem cells [3]. Jiao and colleagues have shown that iN cells could be effectively derived from glioma cells by a combination of Ascl1, Brn2 and Ngn2 [33]. The authors noted that the over-expression of the pro-neural factors dramatically inhibited the proliferation of glioma cells both in vitro and in vivo. In another very recent report, Dirk’s laboratory investigated the molecular stratification of patient-derived glioblastoma cell types, and noted that a subset of glioblastoma stem cells (GSCs) have high levels of Ascl1 [9]. Interestingly, for these Ascl1-high GSCs, inhibition of Notch signaling induced the expression of neuronal markers (bot not astrocyte markers). There is apparently a drive towards neuronal lineage commitment and reduction in self renewal capacity in the GSCs with Notch inhibition by γ-secretase inhibitors, and this response to Notch signaling inhibition was shown to be dependent on Ascl1. Forced expression of Ascl1 alone in Ascl1-low or Ascl1-deleted GSCs using a tetracycline-regulated (Tet-on) construct was sufficient to induce neuronal differentiation. Importantly, forced Ascl1 expression attenuated tumor progression in intracranial xenografts, attesting to its potential in attenuating tumor growth in vivo. As in fibroblasts, Ascl1 mediated neuronal differentiation of GSCs by acting as a pioneer factor, binding and opening new chromatin sites to activate a neurogenic program. These findings suggest that a combination of Ascl1 expression with Notch inhibition in GSCs could potentially halt tumor progression by reducing these from highly proliferative cancer stem cells into one that is neuronal committed and cell cycle exiting. However, the degree of neuronal differentiation of the GSCs in this regard needs to be better defined.

## 5. Targeting CNS Injury and Malignancy with Exogenous Ascl1 Over-Expression

The findings described in the sections above entice the following crude formulation of using Ascl1, in a form of gene therapeutic approach, to curb reactive astroglia proliferation and glioblastoma tumor growth. This forced conversion of unwanted proliferative brain cells into non-dividing neuron-like cells is a contrasting alternative to the more common approach of targeting proliferation drivers or oncogenic drivers, and may have a higher degree of specificity compared to other gene therapeutic approaches such as those that target, for example, more broadly acting micro-RNAs. To begin with, Ascl1 expression mediated by adenoviral-associated vectors or the like should be more widely tested in animal CNS injury and cancer xenograft models for their potential benefits. Other possible alternative modes of delivery include the use of functionalized Ascl1 protein, which could be delivered intracellularly [34], an approach that might be more useful when dealing with tumor masses. In such experiments, measurable beneficial effects may stem from a significant reduction in the proliferating cells concerned, possibly resulting in improved behavioral discovery for CNS injuries, and the prolonging of survival for xenografted animals. The prevalence of cancer stem cells is not limited to glioblastomas, and the conversion of other types of highly proliferative brain cancer cells towards the cell cycle exiting neuronal lineage should be explored. For the cases of CNS injuries, forced expression of Ascl1 may have the added effect of promoting axonal regeneration via the upregulation of factors such as GAP43, which has been demonstrated in a zebrafish optic nerve crash and a rat spinal cord injury model [35]. There is also the possibility of replacing lost neurons by inducing neuronal differentiation of dormant stem or progenitor cells in the CNS [36].

There are a number of immediately notable caveats associated with this strategy. The first, and perhaps most important as far as translational application is concerned, is the efficiency of conversion. Better understanding of the Ascl1 driven differentiation process in different cell types would be necessary to improve differentiation efficiency. As discussed above, reprogramming of reactive astrocytes may work better in certain parts of the CNS, and not in other parts, as the site and environment [31] could influence the neurogenic potential of Ascl1 when administered alone. In these cases, a combination of Ascl1 with other pro-neural factors, such as Sox2 [37], could be attempted. It should also be remembered that Ascl1 is a lineage oncogene in lung and neuroendocrine cancers [38]. The tumorigenic potential of its use in gene therapy should therefore be carefully assessed.

Although astrogliosis and glial scar formation following injury were classically perceived to be detrimental for surviving cells and prevents axonal regeneration, it should be noted that these processes have beneficial aspects [39,40]. Reactive astroglia has a role in glutamate clearance in events of glutamate excitotoxicity [41], regulates GABA neurotransmission and seizures [42], and forms barriers to limit immune inflammation [43]; and scar formation has been recently shown to aid axonal regeneration under certain contexts [44]. Complete elimination of astroglia activation and scar formation upon ischemic or traumatic physical CNS injury may therefore be undesirable. Lastly, it is questionable if the neurons or neuron-like cells converted from astroglia or cancer stem cells would simply remain inert and not perturb normal brain function, even within the relatively non-plastic adult brain structures. There is, of course, the likelihood of collateral neurite growth and perhaps processes of synapsing that could lead to epileptic episodes and psychiatric issues. These confounding issues demands advances in the precision of gene delivery in vivo, and effective reduction of any potential side effects before the approach could become useful in a clinical setting.

## 6. Epilogue

In the preceding paragraphs, the possibility of forced expression of Ascl1 as a potential therapeutic approach for brain injury and cancer is considered. While there are a number of caveats and the idea remains crude at the moment, its efficacy and safety is testable with well-established animal models of stroke [45] and glioma [46]. In view of the devastating effect of stroke and glioblastoma and the dismal paucity of therapeutic options for these conditions, this Ascl1-based strategy should be worth further exploration.
